# Anaplastic Large B-Cell Lymphoma: Cutaneous Presentations

**DOI:** 10.7759/cureus.71430

**Published:** 2024-10-14

**Authors:** Ian Depew, William T Snider, Shane Cook

**Affiliations:** 1 Department of Dermatology, Marshall University Joan C. Edwards School of Medicine, Huntington, USA

**Keywords:** alk-negative alcl, anaplastic large cell lymphoma, brentuximab therapy, cd30-positive lymphoma, chemotherapy treatment, cutaneous involvement, lymph node biopsy, pediatric lymphoma, primary cutaneous alcl, t-cell neoplasm

## Abstract

Anaplastic large cell lymphoma (ALCL) is a rare T-cell lymphoma characterized by CD30 expression. This report describes the case of a 10-year-old female who presented with non-resolving cutaneous lesions initially treated as a bacterial infection. A biopsy confirmed the diagnosis of anaplastic lymphoma kinase-negative (ALK-negative) ALCL with cutaneous and nodal involvement. Further imaging revealed neoplastic uptake in the right lung, and the patient was diagnosed with Murphy stage II ALCL. She began chemotherapy according to established pediatric oncology protocols.

ALCL presents diagnostic challenges due to its non-specific symptoms, which can mimic benign conditions. This case underscores the importance of early biopsy and molecular testing when standard treatments fail. Early recognition and routine examinations, including lymph node assessments and skin biopsies, are critical for improving patient outcomes, as timely diagnosis leads to more effective treatment options and potential remission.

## Introduction

Anaplastic large cell lymphoma (ALCL) is a cancer subtype arising from mature T-cells. The hallmark surface marker for ALCL is CD30 [1]. ALCL can be challenging to diagnose because its symptoms are often vague and can overlap with those of various other diseases. Typically, ALCL manifests in the lymph nodes but can also involve other organs and present with symptoms such as coughing, shortness of breath, swelling, abdominal pain, constipation, loss of appetite, urinary obstruction, leg weakness, and loss of bowel movements. The wide range of symptoms and the presence of different ALCL subtypes affecting various age groups contribute to the difficulty in early detection [2].

There are four subtypes of ALCL: anaplastic lymphoma kinase-positive ALCL, ALK-negative ALCL, primary cutaneous ALCL (pcALCL), and breast implant-associated ALCL [1]. Each subtype has distinct prognostic implications, treatment regimens, and affected populations, complicating both diagnosis and management. For instance, systemic ALK-positive ALCL commonly occurs in younger patients and is characterized by the rearrangement of the ALK gene. It generally has a relatively favorable prognosis, with a five-year survival rate of approximately 70-80% [3]. Systemic ALK-negative ALCL, on the other hand, typically affects older individuals and lacks ALK gene rearrangement, leading to a less favorable prognosis with a five-year survival rate of around 40-60% [3]. pcALCL is confined to the skin and usually has an excellent prognosis with a five-year survival rate exceeding 90%, spreading slowly and being less aggressive [3].

Most lymphomas present with swollen, painless lymph nodes and associated “B” symptoms such as night sweats, fevers, and chills. ALCL can present in this manner but also in various ways depending on the subtype of the ALCL [4]. This report describes the case of a young female patient who presented with a violet papule in her right cubital fossa. Understanding the different manifestations of ALCL is crucial, as diagnosis delays can lead to treatment delays [5].

This case was presented at the West Virginia Dermatological Society Conference on August 9, 2024.

## Case presentation

**Figure 1:**
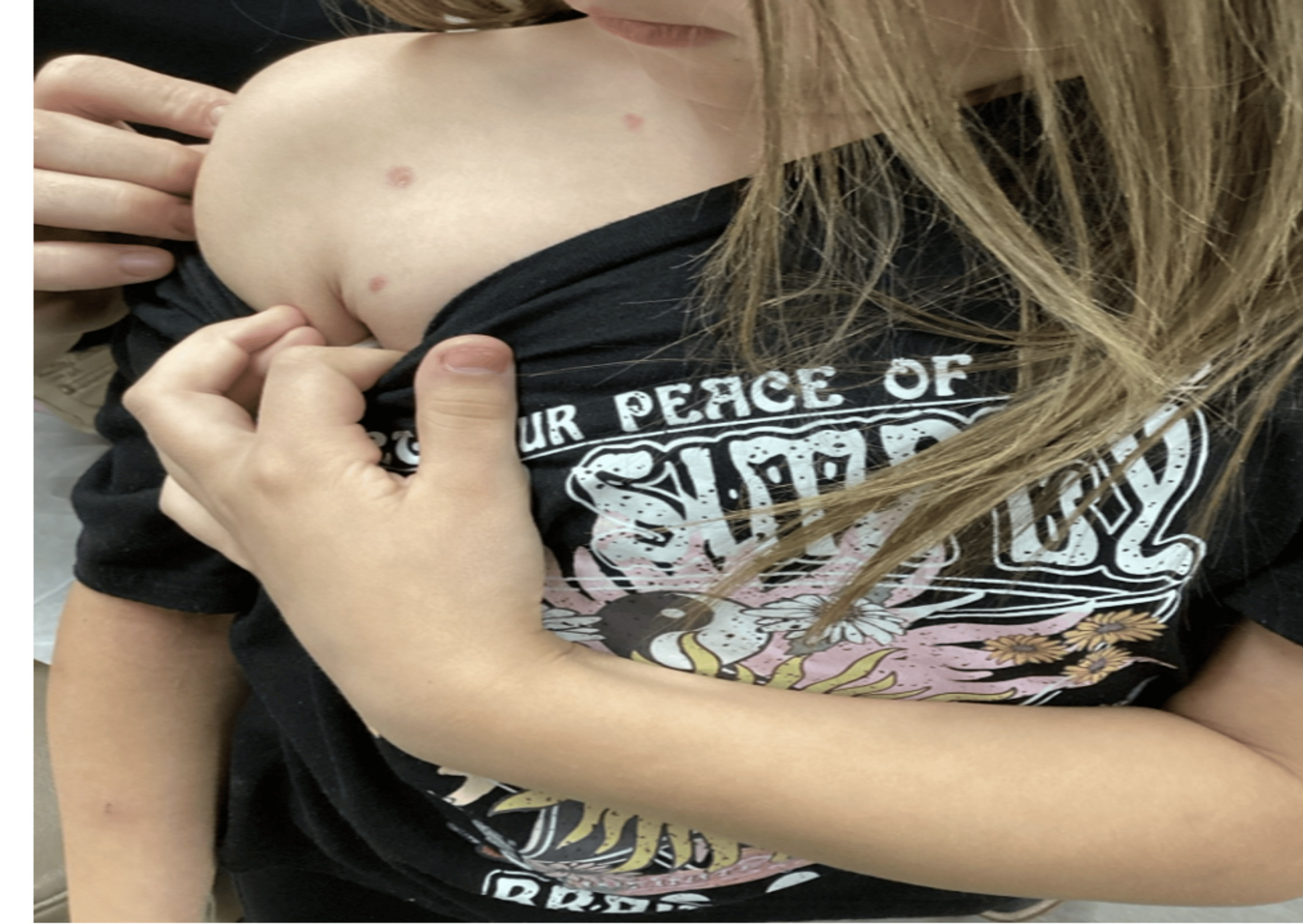
Initial presentation of pink to violet plaques on the right shoulder with similar plaques located throughout the arms, back, and chest area.

**Figure 2:**
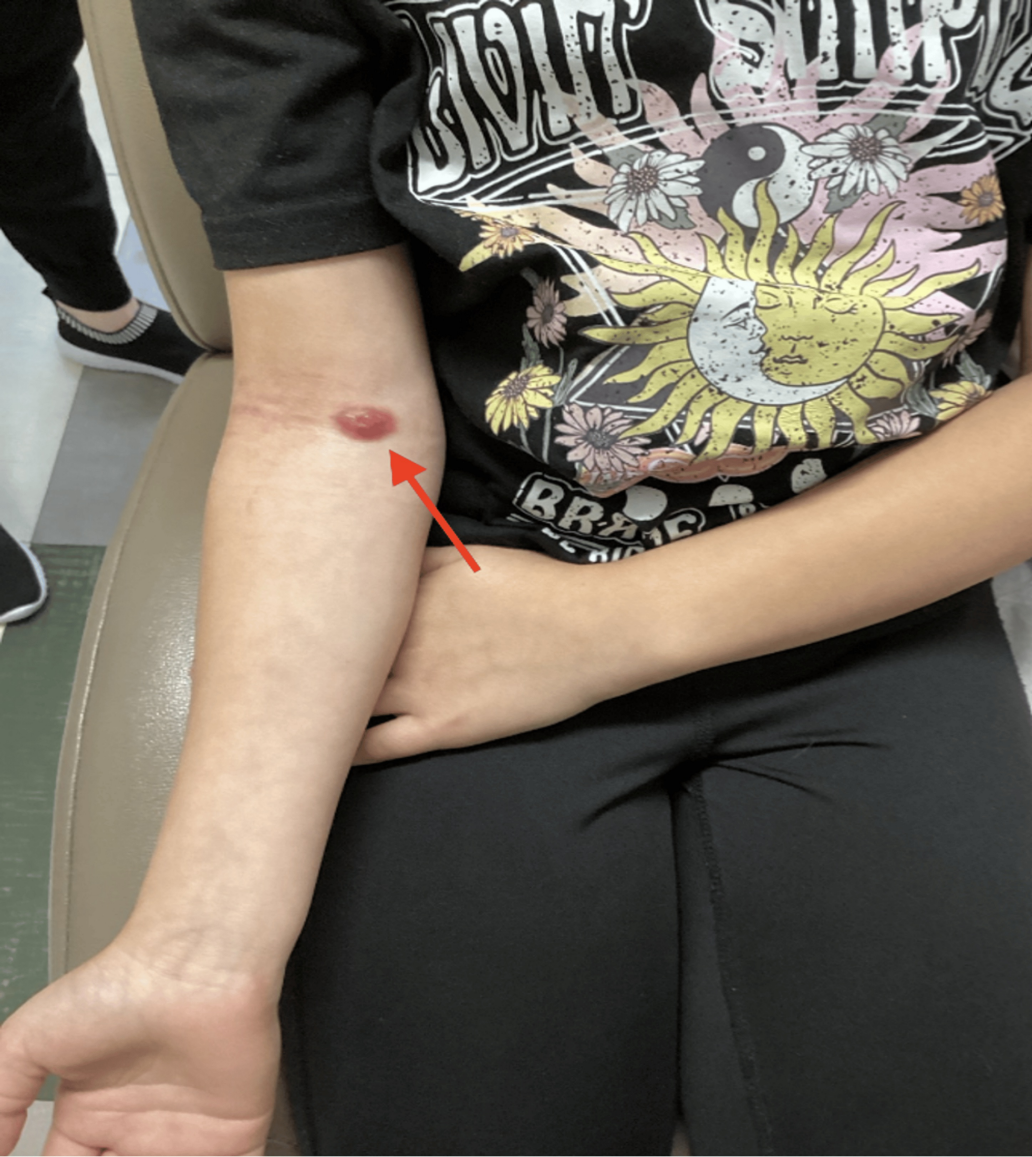
Pink-to-violet plaques on the right antecubital fossa. The red arrow represents the initial punch biopsy site that returned as atypical CD30-positive T-cell lymphoid infiltrate.

**Figure 3:**
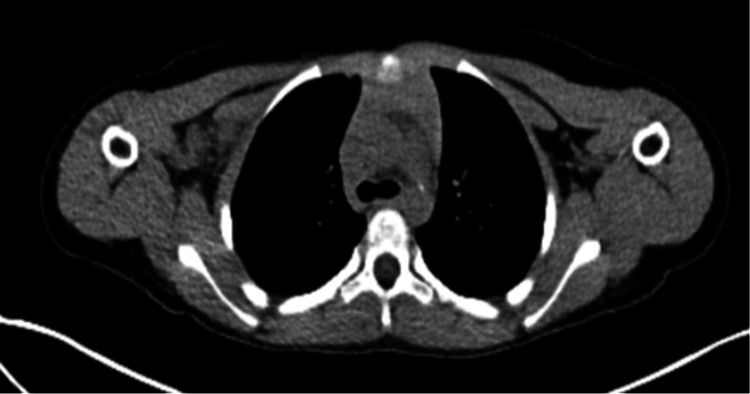
PET CT full-body scan demonstrating right axillary adenopathy and neoplastic uptake in the right lung.

A 10-year-old female initially presented with non-resolving sores on her arms, back, and chest that persisted for one month despite treatment with Bactrim and mupirocin ointment for suspected methicillin-resistant *Staphylococcus aureus* (MRSA) (Figures 1, 2). A culture from a lesion on the right antecubital fossa was negative for MRSA. The patient showed mild improvement with doxycycline therapy, which was discontinued due to gastrointestinal upset, leading to exacerbation and oral intolerance. A biopsy revealed an atypical CD30-positive T-cell lymphoid infiltrate, leading to considerations of lymphomatoid papulosis and ALCL. Molecular testing confirmed T-cell gene rearrangement. Computed tomography (CT) and positron emission tomography (PET) scans demonstrated right axillary adenopathy and neoplastic uptake in the right lung, supporting a diagnosis of ALK-negative ALCL, Murphy stage II, with cutaneous involvement (Figure 3). Following confirmation via biopsy of the right axillary node, the patient underwent bone marrow biopsy and intrathecal chemotherapy. She was subsequently admitted for induction chemotherapy according to the ANHL 12P1 protocol from the Children's Oncology Group.

## Discussion

This patient exhibited a classical presentation of ALCL, but diagnosing ALCL remains challenging due to the common first symptom of skin nodules [6]. The differential diagnosis for ALCL includes other CD30-positive T-cell lymphoproliferative disorders, classical Hodgkin lymphoma, diffuse large B-cell lymphoma, undifferentiated carcinoma, melanoma, and dendritic cell neoplasms [2]. The wide range of possible diagnoses highlights the difficulty in identifying ALCL. Additionally, the age of onset complicates diagnosis, as the average age for ALK-negative ALCL onset is 40-65 years, making it a hard diagnosis in our patient of 12 years of age [7]. ALCL spreads hematogenously and displays large pleomorphic cells, which can mimic other carcinomas and sarcomas, creating histological challenges due to the overlap with various cancers [3]. Distinguishing between the ALCL subtypes requires careful examination of cell markers and clinical presentation.

Biopsy of the nodules is essential for diagnosis, with histology showing large anaplastic and pleomorphic cells with horseshoe nuclei known as "hallmark cells" [8]. Lymph node biopsy reveals sinusoidal infiltration, and necrosis and angioinvasion are common due to the hematogenous spread of ALCL [3]. Immunohistochemistry plays a critical role in diagnosis, with ALCL almost always positive for the CD30 marker [1]. While ALCL can also be positive for ALK, some subtypes are ALK-negative. Epithelial membrane antigen is another marker used to differentiate ALCL from other lymphomas but is less specific [2]. pcALCL is characterized by only cutaneous involvement and responds exceptionally well to surgery and/or radiation, being CD30-positive and ALK-negative [8].

Our patient was confirmed to have ALK-negative ALCL, characterized by CD30 positivity and ALK negativity. This subtype generally has a poorer prognosis with a higher recurrence rate and lower remission rate compared to ALK-positive ALCL [5]. ALK-negative ALCL often presents with advanced staging at diagnosis, as seen in this case with neoplastic uptake in the right lung.

Treatment for ALCL depends on the subtype, disease stage, and patient health factors [7]. The mainstay treatment is a combination chemotherapy regimen, such as cyclophosphamide, doxorubicin, vinblastine, and prednisone (CHOP), which is effective for both ALK-positive and ALK-negative ALCL [9]. Brentuximab vedotin is an antibody-drug conjugate that has become a first-line therapy in treating ALCL. It has been shown to have high response rates in clinical trials of refractory ALCL. It was used as a standalone treatment and had response rates of 53% to 86% in phase 1 and 2 settings, respectively, for relapsed ALCL [10]. Radiation therapy is used in two forms: consolidative for early stage or pcALCL, and palliative for symptom control in relapsed or recurrent cases [11].

The patient was initially treated with a regimen similar to CHOP, including cyclophosphamide, ifosfamide, methotrexate, cytarabine, and dexamethasone, with effective remission induction [9]. If this regimen fails, brentuximab may be considered as ALK-negative ALCL is CD30-positive. Consolidative radiotherapy could also benefit cutaneous lesions. Pediatric ALCL cases with similar presentations have a survival rate of 65-90% with appropriate chemotherapy [12].

## Conclusions

ALCL is a challenging disease to diagnose, especially in its early stages. Its symptoms overlap considerably with other benign and malignant conditions that can mask this disease. A thorough examination, early recognition, and timely use of diagnostic tools such as biopsies and molecular testing are crucial for detecting ALCL. Routine assessments, including lymph node evaluations and further imaging on suspicion, can assist in identifying the disease earlier, improving outcomes. ALCL is not hereditary; therefore, simple examinations and diagnostic tools can detect this pathology before worsening outcomes ensue.
